# Four color B cell immunospot assays for simultaneous detection of all four immunoglobulin classes and subclasses

**DOI:** 10.1186/2051-1426-2-S3-P146

**Published:** 2014-11-06

**Authors:** Richard Caspell, Fredrik Terlutter, Alexey Karulin, Paul V Lehmann

**Affiliations:** 1 Cellular Technology Ltd., Shaker Hts., OH, USA

## Introduction

ELISPOT is the only technique currently available that permits to enumerate antigen-specific B cells providing insights into humoral immune defense. Resting memory B cells can be detected in blood samples after polyclonal stimulation. In contrast, the spontaneous production of immunoglobulins (Ig) by B cells in freshly-isolated peripheral blood mononuclear cells (PBMC) signifies recent and ongoing antigen stimulation in vivo. ELISPOT has thus far been restricted to single color analysis of Ig classes or subclass for detection of Antibody-Secreting Cells. Consequently, a comprehensive study of all four Ig classes or IgG subclasses required fourfold amounts of PBMC, materials and labor. We have developed a 4-color B cell ImmunoSpot assay where all four Ig classes or IgG subclasses are detected simultaneously. This novel technique was applied to the characterization of Human Cytomegalus Virus (HCMV)-specific Ig responses in human subjects with previous exposure to the virus.

## Methods

PBMC were cultured for 4 days with polyclonal B cell activators. On day 4, cells were washed, counted and plated in a low autofluorescence PVDF plate, pre-coated with either anti-human Ig capture antibody for detection of total Ig secretion, or HCMV antigen for detection of HCMV-specific ASC. During a 4h culture, the Ig secreted by the individual B cells was captured on the membrane. The plate-bound human Ig "spots" were visualized using IgG subclass- or Ig class-specific detection reagents, whereby each detection reagent is distinguished from the other 3 reagents through its unique fluorescence. The four-color assays were analyzed using an ImmunoSpot^®^ S6 Analyzer.

## Results/conclusions

The four-color ImmunoSpot assay has identical sensitivity for detecting individual B cells secreting IgG subclasses as the respective single-color assays, and the four fluorochromes can be discerned unambiguously, without spectral overlap. Therefore, four color B cell ImmonoSpot assays are equally suitable for monitoring B cell-mediated immunity as four single-color assays. Applying the test to characterize the HCMV-specific ig response suggest that the same Ig classes and sub-classes are involved in different donors, yet at highly variable ratios. Morover, the spot morphologies showed striking differences amongs the test subjects suggesting the involvement of different B cell affinities: diffuse, "fuzzy" spots indicate relatively low affinity, while tight spots with well-defined borders indicate Ab with high affinity for the antigen. Four color B cell ImmunoSpot assays therefore pemit comprehensively measuring frequencies and affinities of antigen-specific B cells secreting Ig of all four Ig classes and IgG subclasses.

